# Salinity-Modulated Germination and Metabolic Reprogramming in Quinoa Seedlings: Genotype-Dependent Effects on Nutritional and Bioactive Compounds and Prospects for Microgreen Valorization

**DOI:** 10.3390/plants15203108

**Published:** 2026-10-11

**Authors:** Rahma Goussi, Rim Ben Youssef, Hatem Ben Jouira, Chiraz Friji, Sawsen Selmi, Halima Khaled, Arafet Manaa

**Affiliations:** 1 Laboratory of Extremophile Plants, Centre of Biotechnology of Borj Cedria, P.O. Box. 901, Hammam-Lif 2050, Tunisia; rahma.goussi@cbbc.rnrt.tn (R.G.); rim.benyoussef@hotmail.com (R.B.Y.); hatem.benjouira@cbbc.rnrt.tn (H.B.J.); chiraz.friji@cbbc.rnrt.tn (C.F.); selmisawsencbbc@gmail.com (S.S.); 2 Saharan Bio-Resources Laboratory, Safeguarding and Valorization, Kasdi Merbah Ouargla University, Ouargla 30000, Algeria; khhalima2004@gmail.com

**Keywords:** quinoa, salinity stress, seed germination, seedlings, secondary metabolites, nutritional quality, microgreens

## Abstract

Germination is highly sensitive to abiotic stresses such as salinity, which can impair seedling establishment and seed reserve mobilization. Quinoa is a promising crop due to its salinity tolerance and high nutritional value. This study evaluated the effects of NaCl-induced salinity on germination performance, metabolic responses, and nutritional quality of three quinoa seed lots representing two varieties, Regalona (Q1) and *Amarilla sacaca* (Q2, Q3). Salinity significantly reduced germination rate and kinetics, as well as radicle and shoot growth, with the strongest effects at 500 mM NaCl. During the germination process, starch and protein contents decreased, reflecting reserve mobilization, while vitamin E increased under moderate salinity, particularly in Q1 and Q2, but declined under severe stress. Secondary metabolites, including phenolics, flavonoids, tannins, and saponins, exhibited strong genotype- and stress-dependent variation. HPLC-DAD identified gallic, syringic, vanillic, caffeic, and protocatechuic acids, as well as rutin, quercetin, and kaempferol, with marked differences between raw seeds and seedlings. At moderate salinity (300 mM NaCl), TFC increased by 22% and 42% in Q1 and Q2, respectively, relative to RS, whereas Q3 showed a 45% reduction. Under high salinity (500 mM NaCl), TFC decreased by 83% and 15% in Q1 and Q2, respectively, while Q3 exhibited a 35% increase relative to RS. These contrasting responses highlight genotype-dependent changes in flavonoid accumulation and reflect distinct metabolic plasticity among quinoa genotypes. Overall, these findings support quinoa as a promising functional microgreen crop for producing nutrient- and bioactive-rich foods under controlled conditions.

## 1. Introduction

Originating from the Andean region of South America, *Chenopodium quinoa* Willd. is an annual herbaceous plant, whose exceptional nutritional value and ability to adapt to diverse environmental conditions have contributed to growing interest in its potential as a promising crop for sustainable food production [1,2]. Quinoa is a rich source of protein with high quality providing a balanced profile of essential amino acids [3]. Quinoa seeds are also rich in dietary fiber, and essential minerals such as magnesium, iron, manganese, copper, calcium, and phosphorus. The seeds contain 5–11% oil, characterized by a high percentage of polyunsaturated fatty acids (especially omega 3, 6, 9) and a favorable ω-6/ω-3 ratio essential for human growth and development (brain, muscles), as well as very high levels of vitamin E or tocopherols (α, β, γ and δ) and tocotrienols (α, β, γ and δ) with known biological activity, essential for reproduction and growth of mammals including humans [4]. In addition to its remarkable nutritional profile, quinoa is well known for its gluten-free grains, low glycemic index owning to its high-quality carbohydrates [5]. Furthermore, quinoa seeds contain a diverse range of bioactive compounds, including secondary metabolites that can influence nutrient bioavailability and exhibit important biological activities [6]. These compounds have attracted considerable interest for their antioxidant properties and potential applications in the pharmaceutical and nutraceutical fields [7]. Among the major bioactive compounds found in quinoa are polyphenols and flavonoids, which are widely recognized for their antioxidant and health-promoting properties, as well as tannins and saponins, which are traditionally considered antinutritional factors but may also exhibit beneficial biological activities [8].

Quinoa is a facultative halophyte that belongs to the Amaranthaceae family, which is well known for its high proportion of halophytic species [9]. This species is highly adaptable and can tolerate a wide range of agroecological conditions [10] and is highly resistant to various abiotic stresses, including salinity [11,12], drought [13,14], frost [15], and heat [16]. Thanks to this remarkable resilience, quinoa is considered a suitable alternative crop for enhancing global food security and has attracted significant international attention, particularly following the declaration of 2013 as the International Year of Quinoa by the United Nations (FAO). As a result, its cultivation has expanded rapidly from only eight countries in 1980 to over 150 countries in recent years [17]. From a commercial perspective, quinoa is a highly versatile crop, utilized not only for its grains but also as a green vegetable, including sprouts, seedlings, and microgreens, which are increasingly popular in healthy diets. These innovative culinary products can be produced quickly, easily, and cost-effectively due to minimal equipment and material requirements and a rapid development process ranging from a few days for sprouts to approximately two weeks for microgreens [18]. Quinoa greens (sprouts, leaves and microgreens) have emerged as promising healthy vegetables and are increasingly being explored for the development of functional and value-added food products [19]. Compared with mature quinoa leaves, microgreens can provide a concentrated source of nutritionally and biologically relevant compounds, while both quinoa seeds and green tissues have attracted considerable attention because of their diverse bioactive constituents, including dietary fiber, polyphenolics, bioactive peptides, saponins, and other antioxidant compounds. In particular, quinoa microgreens are rich in soluble dietary fiber [20] and can contain substantially higher mineral concentrations than mature quinoa grains, with reported calcium and potassium contents reaching 1535 and 8769 mg/100 g, respectively, compared with 148.7 and 1475 mg/100 g in mature grains [19]. They are also characterized by an enrichment in lipid-soluble antioxidants, including tocopherols and β-carotene, which contribute to their antioxidant potential and may increase their interest for nutraceutical and functional food applications [21]. However, despite their nutritional and functional potential, the nutritional and antinutritional content of raw seeds and seedling depends on several factors, including species, cultivar, environmental conditions, and the duration of germination, storage, and processing [22,23,24]. In this context, several studies have shown that stress conditions such as salinity, temperature, light, heavy metals, and micronutrients can have both positive and negative effects on seedling growth parameters, including germination percentage, germination rate, seedling vigor, shoot and root length, fresh and dry weight, and overall biomass [24]. For instance, previous report [25] demonstrated that increasing salinity levels significantly reduced germination and seedling growth across 25 quinoa genotypes. Germination declined by approximately 60% as salinity increased, indicating substantial sensitivity to salt stress. At the highest salinity level (30 dS m^−1^), no germination was observed in any genotype, demonstrating the severe inhibitory effect of extreme salinity conditions.

Furthermore, the performance and quality of seedlings or germinated seeds are strongly influenced by various abiotic stress factors, which not only affect morphological parameters as described above but also the accumulation of bioactive compounds, including secondary metabolites and antioxidants [24]. Previous report on rapeseed (*Brassica napus* var. *oleifera* Del.) demonstrated that salinity enhanced the antioxidant capacity of rapeseed by increasing the free fractions of total phenolics, non-flavonoids, tannins, and phenolic acids [26]. In addition, moderate salinity levels (25–50 mM NaCl) produced the greatest increase in phenolic compounds and antioxidant activity with only minor effects on seedlings growth. Previous studies on the halophyte species *Glaux maritima* have shown that moderate to high salinity stimulates phenolic metabolism [27]. The highest concentrations of total phenolics, flavonoids, and hydroxycinnamic acids were observed at 50–100, 75–400, and 200–300 mM NaCl, respectively. Salinity also promoted the accumulation of key phenolic compounds such as rutin, hyperoside, and catechin.

Collectively, these findings suggest that the controlled application of abiotic stress during seedling establishment may be exploited as a priming strategy to modulate growth and enhance the nutritional and functional quality of raw seeds or seedlings by stimulating the accumulation of bioactive compounds and increasing antioxidant potential. However, information remains limited on the responses of quinoa seedlings to abiotic stress, particularly regarding the accumulation of secondary metabolites and the mobilization of storage compounds during germination under saline conditions. Therefore, the present study aimed to investigate the effects of NaCl-induced salinity on quinoa germination, with particular emphasis on the accumulation and mobilization of primary and secondary metabolites in developing seedlings. This work provides preliminary insights into the physiological and biochemical responses of quinoa during early development and highlights the potential of controlled salinity stress for the valorization of quinoa as a functional microgreen crop.

## 2. Results

### 2.1. Effect of Salinity on Germination and Early Seedling Growth in Quinoa Genotypes

The results presented in Figure 1a showed that increasing NaCl concentration exerts a significant inhibitory effect on seed germination in all quinoa seed lots (Q1, Q2, and Q3), with a pronounced variability in their responses. Under moderate salinity (300 mM NaCl), the germination rate of Q1 decreased to about 60% compared to the control. In Q2, germination rate remained relatively high showing only a moderate decrease of about 20%, which suggests a higher tolerance to salt stress compared to other genotypes. In contrast, Q3 exhibited a significant decline to around 45% under 300 mM NaCl, as compared to control. This inhibitory effect became more pronounced at 500 mM NaCl, where germination rate in genotypes Q1 and Q3 dropped drastically to approximately 95% and 90%, respectively, indicating almost complete suppression and high susceptibility to severe salinity. However, similar to the 300 mM condition, Q2 maintained a comparatively high germination rate relative to the other genotypes. Only a reduction of about 18% and 33% was recorded in this genotype under 300 mM and 500 mM NaCl, respectively, compared to the control.

In addition, the decrease in germination rate was accompanied by a significant increase in mean germination time (T_1/2_) in all quinoa genotypes (Figure 1b). In fact, T_1/2_ generally increased with increasing salt concentration in the growth medium, although some inter-genotypic differences were observed. Under control conditions (0 mM NaCl), Q2 exhibited the lowest T_1/2_ (≈35 h), indicating faster germination compared to Q1 (≈57 h) and Q3 (≈68 h). At 300 mM NaCl, T_1/2_ increased in all genotypes, reaching approximately 78 h, 55 h, and 88 h for Q1, Q2, and Q3, which correspond to an increase of about 37%, 57%, and 29% for Q1, Q2, and Q3, respectively, compared to control conditions. This effect became more pronounced at 500 mM NaCl, where T_1/2_ further increased to around 99 h, 58 h, and 111 h in Q1, Q2, and Q3, respectively. These values represent an increase of approximately 74%, 66%, and 63% for Q1, Q2, and Q3, respectively, relative to the control.

Despite this general delay in germination, genotype Q2 consistently maintained the lowest T_1/2_ across all salinity levels, indicating faster germination and better tolerance to salt stress. In contrast, Q3 showed the highest T_1/2_ values, reflecting a greater sensitivity and slower germination under saline conditions, while Q1 displayed intermediate behavior.

Salt stress, significantly, affected both radicle and shoot growth in all quinoa genotypes, with a significant genotype-dependent response (Figure 1c,d). Under control conditions (0 mM NaCl), Q1 exhibited the highest radicle (12 cm) and shoot length (7 cm), followed by Q2 and Q3. With increasing salinity (300 mM NaCl), radicle length showed a significant decrease in Q1 (67%) and Q3 (29%), whereas a significant increase (25%) was observed in Q2. At high salinity (500 mM NaCl), the inhibitory effect became more prominent and radicle length decreased drastically in Q1 (83%), Q2 (38%) and Q3 (57%) relative to the control. A similar trend was observed for shoot length at 500 mM NaCl, with significant decreases of approximately 70%, 35% and 47% in Q1, Q2, and Q3, respectively.

### 2.2. Effects of Salinity on Nutritional Composition of Quinoa Seedlings

#### 2.2.1. Protein Content

Protein content in raw seeds (RS) and quinoa seedlings varied significantly among varieties and salt treatments (Figure 2a). In raw seeds, protein levels were highest across all seed lots, with Q2 showing the greatest content, followed by Q3 and Q1. Upon germination, regardless of salinity level (0, 300, or 500 mM NaCl), protein content decreased significantly in all varieties, although Q1 consistently retained higher levels than Q2 and Q3. Under non-saline conditions (0 mM NaCl), protein content declined by approximately 29%, 53%, and 68% in Q1, Q2, and Q3, respectively, compared to raw seeds. Under 300 mM NaCl, the reduction became more pronounced, ranging from 71% to 76% across all genotypes. At the highest salinity level (500 mM NaCl), protein content showed the greatest decrease in all genotypes, with reductions of around 64% in Q1 and up to 94% in both Q2 and Q3 as compared to raw seeds.

#### 2.2.2. Soluble Sugars and Starch Content

Soluble sugar content in quinoa RS and seedlings varied markedly among quinoa seed lots (Q1, Q2, and Q3) and salt treatments (0, 300, and 500 mM NaCl) (Figure 2b). Variety Q1 consistently exhibited the highest sugar levels at all growth stages (raw seeds and seedlings). Under non-stressed conditions (0 mM NaCl), Q1 showed the highest soluble sugar content (4.9 g/100 g), followed by Q3 (~3.0 g/100 g) and Q2 (~2.6 g/100 g), all of which represented substantial increases compared with raw seeds across genotypes. These increases correspond to approximately 194%, 80%, and 138% for Q1, Q2, and Q3, respectively, from RS to 0 mM NaCl, indicating a strong metabolic activation of carbohydrate mobilization during germination under optimal conditions.

Salt stress significantly reduced sugar accumulation in all varieties as compared to the control (0 mM NaCl). At 300 mM NaCl, sugar content significantly decreased by approximately 14%, 35% and 33% in Q1, Q2 and Q3, respectively, relative to the control. This negative effect was more prominent at 500 mM NaCl, where sugars content dropped considerably by about 75%, 65%, and 67% in Q1, Q2 and Q3, reaching near-minimal levels as for the raw seeds.

Regarding starch content, Q2 exhibited the highest level in raw seeds (2.2 g/100 g), followed by Q1 (~1.6) and Q3 (~1.4) (Figure 2c). After germination under control conditions (0 mM NaCl), starch content decreased markedly in all varieties, with reductions of approximately 72% in Q1, 83% in Q2, and 40% in Q3 compared to raw seeds. Under salinity conditions, starch content increased progressively with increasing NaCl concentration, particularly in Q1 and Q2. At 300 mM NaCl, starch levels increased by about 56% in Q1 and 220% in Q2 relative to the control (0 mM), followed by a slight additional increase at 500 mM. In contrast, Q3 showed a different pattern, with a slight decrease (20%) at 500 mM compared to the control.

#### 2.2.3. Vitamin E Content 

Vitamin E content (assessed as α-tocopherol) showed a clear stress-dependent pattern across the three quinoa seed lots (Q1, Q2, and Q3), with relatively low levels detected in raw seeds (RS) (Figure 2d). Among the raw seeds, genotype Q1 exhibited the highest vitamin E content (2.1 mg/100 g DW). During seedling development under control conditions (0 mM NaCl), vitamin E content increased significantly in all genotypes compared with raw seeds, with increases of approximately 95% in Q1, 168% in Q2, and 200% in Q3.

Under moderate salinity (300 mM NaCl), a further significant stimulation of α-tocopherol content was observed in Q1 and Q2, increasing by approximately 46% and 21%, respectively, relative to the control seedlings. In contrast, Q3 exhibited a 33% decrease under the same conditions. However, exposure to severe salinity (500 mM NaCl) resulted in a pronounced reduction in α-tocopherol levels in all varieties, with decreases of approximately 68%, 60%, and 82% in Q1, Q2, and Q3, respectively, compared with the control seedlings. Among the studied genotypes, Q1 displayed the highest α-tocopherol content (6 mg/100 g DW) under moderate salinity, suggesting a greater degree of salt tolerance, followed by Q2.

### 2.3. Effects of Salinity on Bioactive and Antinutritional Compounds

#### 2.3.1. Saponin Content

The saponin content varied significantly among the quinoa seed lots (Q1, Q2, and Q3), developmental stages (raw seeds and seedlings), and NaCl treatments (Figure 3a). Overall, the highest saponin content was observed in raw seeds compared with the seedling stage, regardless of salinity conditions. In raw seeds, genotype Q3 exhibited the highest saponin content (4.2 mg/g DW), followed by Q2 (3.3 mg/g DW) and Q1 (1.4 mg/g DW). Compared with Q1, the saponin content was approximately 136% higher in Q2 and 200% higher in Q3. At the seedling stage under control conditions (0 mM NaCl), saponin levels decreased significantly in all varieties relative to their respective raw seeds. The reduction reached approximately 50%, 67%, and 76% in Q1, Q2, and Q3, respectively.

Under moderate salinity (300 mM NaCl), the response differed among varieties. In Q1, saponin content reached approximately 1 mg/g DW, representing an increase of 38% compared with the control seedlings (0 mM). This stimulatory effect was more pronounced in Q2, where saponin content increased to 2.3 mg/g DW, corresponding to an increase of about 109% relative to the control seedlings. In contrast, Q3 showed a further decline in saponin content to nearly 0.7 mg/g DW, which was approximately 40% lower than the control.

At high salinity (500 mM NaCl), Q1 exhibited the highest saponin accumulation among the treated seedlings, reaching approximately 1.45 mg/g DW, which corresponded to an increase of about 107% relative to the control (0 mM) and was slightly higher than its raw seed value. In Q2, saponin content decreased slightly compared with the 300 mM treatment, reaching around 20%; however, it remained approximately 85% higher than the control seedlings. In contrast, Q3 showed no significant change in saponin content under high salinity compared with the control treatment.

#### 2.3.2. Tannins Content

Figure 3b showed a clear genotype-dependent and salinity-responsive pattern in tannin accumulation across quinoa seedlings (Q1, Q2, Q3) relative to raw seeds (RS). In the baseline state (RS), tannin content was highest in Q3 (~470 mg CE/100 g DW), followed by Q2 (~330 mg CE/100 g DW), and lowest in Q1 (~130 mg CE/100 g DW), showing an initial ranking of Q3 > Q2 > Q1. After germination under control conditions (0 mM NaCl), tannin levels changed considerably, and increased in Q1 to 350 mg CE (171% relative to RS), whereas it decreased in Q2 to 270 mg/100 g (18% reduction), and decreased in Q3 considerably to 180 mg CE (62% reduction). This indicates that developmental activation under non-saline conditions strongly enhances tannin accumulation in Q1, while leading to moderate and severe reductions in Q2 and Q3, respectively.

Under moderate salinity (300 mM NaCl), tannin content showed a genotype-dependent response, with increases of approximately 34%, 45%, and 20% in Q1, Q2, and Q3, respectively, compared to control seedlings (0 mM NaCl). At high salinity (500 mM NaCl), a significant decrease in tannin levels was observed in Q1 and Q2, with reductions of about 53% and 11%, respectively. In contrast, genotype Q3 exhibited a further increase of 60% compared to the control.

#### 2.3.3. Total Phenolics and Flavonoids Content (TPC and TFC)

The variation in total flavonoid and phenolic content among seed lots, development stages and salinity conditions are shown in Figure 3c,d, respectively. In raw seeds, Q2 exhibited the highest TFC (260 mg QE), followed by Q1 (205 mg QE) and Q3 (175 mg QE). After germination under non-saline conditions (0 mM NaCl), TFC decreased significantly in all varieties, with reductions of approximately 31%, 67% and 78%, respectively in Q1, Q2, and Q3 relative to RS. Under 300 mM NaCl, TFC increased considerably in Q1 and Q2, reaching about 250 and 370 mg QE/100 g, corresponding to increases of nearly 22% and 42%, respectively, compared with RS, whereas Q3 still showed a reduction of about 45%. At high salinity (500 mM NaCl), contrasting varietal responses were observed: TFC significantly declined in two genotypes Q1 (83%) and Q2 (15%), while Q3 exhibited an increase of approximately 35% relative to RS.

Similarly, TPC was highest in RS, particularly in Q2 (350 mg GAE/100 g DW), whereas Q1 and Q3 showed similar levels (250 mg GAE/100 g DW). Germination at 0 mM NaCl caused substantial decreases in TPC in all varieties, with reductions ranging between 82% and 89% compared with RS. However, salinity treatment increased TPC in most quinoa varieties compared with the non-saline condition (0 mM NaCl). At 300 mM NaCl, TPC increased in Q1 (+191%) and in Q3 (+67%), whereas Q2 showed a decrease about 21%. At 500 mM NaCl, TPC also increased, particularly in Q1 (+29%) and Q3 (+18%) compared to control seedlings.

### 2.4. Two-Way ANOVA and Pearson Correlation Analysis

The two-way ANOVA analysis (Table 1) showed that salinity conditions significantly affected most of the evaluated traits, while genotype effects and, particularly, genotype × salinity interactions contributed substantially to the observed variability. In fact, significant G × E interactions were detected for germination rate (*p* = 0.0012), radicle length (*p* < 0.001), starch (*p* < 0.001), sugars (*p* < 0.001), proteins (*p* < 0.001), vitamin E (*p* = 0.026), tannins (*p* = 0.012), saponins (*p* = 0.0112), TFC (*p* = 0.0048), and TPC (*p* = 0.0035). Notably, the magnitude of the F-values and the significance levels indicated that the relative importance of the factors differed among traits. For starch and proteins, both genotype and genotype × salinity interactions showed highly significant effects, suggesting that their responses to salinity were strongly genotype-dependent. Similarly, tannins and saponins exhibited significant genotype and genotype × salinity effects, indicating differential responses among genotypes under the different salinity conditions. For TFC and TPC, the genotype × salinity interaction was also highly significant, highlighting the importance of the specific combination of genotype and salinity condition in determining these traits.

The Pearson correlation analysis provided further evidence of coordinated changes among growth, primary metabolites, and bioactive compounds (Figure 4). Germination rate was strongly and positively correlated with radicle length (r = 0.89, *p* < 0.05) and vitamin E (r = 0.79, *p* < 0.05), and moderately correlated with sugars (r = 0.62, *p* < 0.05), indicating that successful germination and early seedling development were associated with efficient metabolic activity and antioxidant accumulation. Conversely, germination rate was negatively correlated with starch (r = −0.58, *p* < 0.05), saponins (r = −0.47, *p* < 0.05), and TPC (r = −0.63, *p* < 0.05). Radicle length showed a similar positive association with sugars (r = 0.58, *p* < 0.05) and vitamin E (r = 0.65, *p* < 0.05), but was strongly negatively correlated with TPC (r = −0.72, *p* < 0.05), suggesting a potential trade-off between seedling growth and phenolic accumulation under stress.

Among the biochemical traits, TPC was strongly and positively correlated with starch (r = 0.82, *p* < 0.05) and proteins (r = 0.76, *p* < 0.05), while TFC was positively associated with tannins (r = 0.54, *p* < 0.05) and saponins (r = 0.36, *p* < 0.05). Sugars were also strongly correlated with vitamin E (r = 0.73, *p* < 0.05), supporting the coordinated regulation of carbon metabolism and antioxidant responses during seedling development.

Correlation analysis revealed that saponin content was positively associated with starch (r = 0.71), total phenolic content (r = 0.63), protein content (r = 0.51), tannins (r = 0.44), and total flavonoid content (r = 0.36) (Figure 4). In contrast, saponin content showed significant negative correlations with germination percentage (r = −0.47) and root length (r = −0.48), indicating that elevated saponin levels may adversely affect seed germination and early seedling growth (Figure 4). Tannins, as polyphenolic compounds, contributing to ROS scavenging and cellular protection, showed a positive correlation with saponins (r = 0.44) and vitamin E (r = 0.30), suggesting coordinated regulation of antioxidant-related metabolites.

### 2.5. Identification of Individual Phenolic and Flavonoid Compounds in Quinoa Seedlings Using HPLC-DAD

The identification of individual phenolic and flavonoid compounds under different conditions present in the hydro-ethanolic fraction of seeds from the three-quinoa seed lots is shown in Table 2. The individual phenolic and flavonoid compounds in quinoa seedlings varied significantly among the genotypes under raw seed (RS), control (0 mM NaCl), and salinity treatments (300 and 500 mM NaCl). In Q1, the transition from RS to control conditions (0 mM) resulted in increases in gallic acid (17.9%), syringic acid (33.3%), rutin (58.8%), and quercetin (384.6%), whereas vanillic acid slightly decreased by 9.5%.

Under 300 mM NaCl, Q1 exhibited further stimulation of several metabolites compared with the control, including gallic acid (+52.5%), syringic acid (+9.7%), rutin (+174.1%), quercetin (+25.2%), vanillic acid (+33.3%), and caffeic acid (+100%). However, severe salinity (500 mM) considerably reduced most compounds relative to control seedling, with reduction of 57.6% for gallic acid, 75.0% for syringic acid, 11.1% for rutin, 50.0% for quercetin, 27.9% for vanillic acid, and 26.3% for caffeic acid.

In genotype Q2, control seedlings showed slight reductions in gallic acid (−3.9%) compared with RS, while syringic acid, quercetin, protocatechuic acid, and caffeic acid increased by 30.1%, 146.3%, 2.8%, and 19.8%, respectively. Exposure to 300 mM NaCl caused only slight reduction in gallic acid and syringic acid, whereas quercetin increased by 10.9%. In contrast, protocatechuic acid and caffeic acid decreased by 51.7% and 8.3%, respectively. At 500 mM NaCl, all detected compounds declined considerably compared with the control seedling, including gallic acid (−67.6%), syringic acid (−74.9%), quercetin (−46.5%), protocatechuic acid (−79.6%), and caffeic acid (−73.6%).

For Q3, the control treatment (0 mM) enhanced most compounds relative to RS, with increases of 5.7% in gallic acid, 35.8% in syringic acid, 139.1% in rutin, 121.2% in quercetin, 414.3% in kaempferol, and 10.8% in caffeic acid, while vanillic acid and protocatechuic acid slightly decreased by 7.6% and 2.3%, respectively. Under moderate salinity (300 mM), rutin and vanillic acid further increased by 58.2% and 45.1%, respectively, whereas gallic acid, syringic acid, quercetin, protocatechuic acid, kaempferol, and caffeic acid showed slight to moderate reductions ranging from 1.1% to 49.3% compared with the control seedling.

Severe salinity (500 mM) substantially suppressed metabolite accumulation in Q3, causing reductions of 52.7% in gallic acid, 71.3% in syringic acid, 61.0% in quercetin, 45.9% in vanillic acid, 77.2% in protocatechuic acid, 70.8% in kaempferol, and 68.8% in caffeic acid relative to the control.

Overall, moderate salinity (300 mM) stimulated the accumulation of several phenolic and flavonoid compounds, particularly in Q1 and Q3, whereas severe salinity (500 mM) markedly reduced most metabolites across all quinoa genotypes.

## 3. Discussion

Seed germination represents one of the most critical stages in the plant life cycle and is strongly influenced by environmental factors such as temperature, oxygen availability, humidity, and salinity [28,29]. Successful germination depends primarily on water uptake by seeds, which activates metabolic pathways involved in embryo growth and reserve mobilization [30]. Salinity is considered one of the major abiotic stresses limiting seed germination and seedling establishment because high salt concentrations can disrupt osmotic balance, impair nutrient uptake, and alter metabolic activities [31,32]. During germination, the activation of metabolic processes leads to the mobilization of seed reserves and to significant changes in the accumulation of bioactive compounds. These physiological modifications can influence the nutritional quality of the emerging seedlings compared with raw seeds, and the magnitude of these changes may vary according to genotype and environmental conditions [33]. In this context, the present study aimed to investigate the variability among quinoa seed lots and their responses to NaCl-induced salinity during germination, with particular emphasis on starch mobilization and the accumulation of sugars, proteins, phenolic compounds, flavonoids, tannins, and saponins.

Our results showed that salinity negatively affected the germination parameters (germination rate and T_1/2_) of all quinoa seed lots, particularly at high salinity level (500 mM NaCl). The observed reduction in germination rate, radicle and shoot growth indicates that salt stress delays or inhibits the metabolic processes required for embryo growth. These findings are consistent with previous studies reporting significant reductions in quinoa seed germination under saline conditions [25,34], with the magnitude of reduction increasing progressively with salinity in all tested seed lots, reaching complete germination inhibition at high salinity levels (up to 30 dS m^−1^). Germination inhibition under saline conditions may be associated with both osmotic stress and ion toxicity caused by Na^+^ and Cl^−^ accumulation, which reduce seed imbibition [35]. Consequently, these effects limit water uptake by seeds, impair the initiation of early germination processes, and prevent the emergence of the radicle from the seed coat [36,37]. Despite the overall negative effect of salinity on germination parameters, seed lot Q2 exhibited greater tolerance compared with Q1 and Q3 (Figure 1). Q2 maintained higher germination percentage and faster germination kinetics under saline conditions, indicating a better capacity to withstand osmotic and ionic stress during the early stages of development. The superior performance of Q2 under salt stress may be related to an efficient ion homeostasis system, limiting the toxic accumulation of Na^+^ and Cl^−^ in embryonic tissues while maintaining adequate K^+^ levels necessary for metabolic activities [35]. Furthermore, better mobilization of seed reserves and maintenance of enzymatic activities involved in germination may contribute to the improved germination capacity observed in Q2. Similar genotypic differences in salinity tolerance during quinoa germination have been reported in previous studies [38].

However, it is important to note that Q2 and Q3 correspond to the same quinoa variety, *Amarilla sacaca*, but were produced under different propagation environments and geographical conditions. Therefore, the contrasting responses observed between Q2 and Q3 should be interpreted cautiously and should not be considered as evidence of genetic variation alone. Differences in the maternal environment during seed development, including temperature, water availability, soil salinity, nutrient status, and other environmental factors, can influence seed maturation, reserve accumulation, hormonal balance, antioxidant capacity, and the physiological quality of the resulting seeds. Such environmental effects may persist after seed maturation and influence germination behavior and seedling responses to subsequent stresses, a phenomenon that may be associated with stress memory [39]. These effects can arise from changes in seed reserves, hormonal balance, antioxidant capacity, and other physiological or epigenetic mechanisms established during seed development. Consequently, the different responses of Q2 and Q3 to salinity may partly reflect differences in their seed-production environments rather than genetic differences. This phenomenon, often referred to as maternal environmental or seed-production effects, can contribute to differences in seed vigor and stress responsiveness even within the same genetic background. Thus, the contrasting salt responses of Q2 and Q3 highlight the importance of considering not only genotype but also seed-production environment when evaluating salinity tolerance in quinoa.

Beyond their effects on germination performance, saline conditions may also influence the biochemical and nutritional composition of seedlings by altering metabolic activities associated with reserve mobilization and the synthesis of bioactive compounds [24]. Therefore, our results on the evaluation of the nutritional quality of raw seeds and seedlings under salinity stress during early developmental stages showed significant variations in nutritional and biochemical traits according to genotype, germination status, and salinity level. In fact, the inhibition of germination under saline conditions may be associated with disturbances in reserve mobilization, particularly starch degradation. Starch is the principal storage carbohydrate in seeds and constitutes the main energy source required for embryo development during germination and its mobilization depends on hydrolytic enzymes such as α-amylase, whose activities are known to decline under saline conditions [40]. In this study, starch content decreased during germination compared with raw seeds, indicating active reserve mobilization to sustain seedling growth. However, under salinity treatments, starch degradation was altered in a concentration- and genotype-dependent manner. The decrease in starch under salt stress could result from delayed water uptake and reduced enzymatic activity caused by NaCl accumulation. Similar observations were described in wheat, rice, and quinoa, where salinity limited starch mobilization and consequently reduced germination efficiency [41]. Moreover, the decrease in starch content in seedling under non-saline conditions confirms that germination stimulates the hydrolysis of starch reserves to provide sugars and energy for the developing embryo. In our case, despite remaining lower than in raw seeds, starch content under high salinity tended to partially recover in genotype Q1 and, more markedly, in Q2, which showed the greatest stability among the tested genotypes. In contrast, Q3 appeared more sensitive under severe salinity stress, exhibiting a stronger reduction in starch levels. These results highlight significant varietal differences in starch utilization and metabolic adjustment in response to salinity, suggesting that Q2 may possess a more efficient balance between reserve mobilization and stress adaptation during early seed development.

Germination is characterized by the activation of proteolytic enzymes that hydrolyze storage proteins into amino acids, which are subsequently utilized for de novo synthesis of structural and functional proteins required for seedling growth [42]. Consequently, the observed reduction in protein content during germination across all quinoa genotypes under both control and saline conditions, as well as from raw seeds to seedlings, can be attributed to the intense metabolic reprogramming that occurs during seedling development. The more pronounced decline under salinity, particularly at 500 mM NaCl, suggests that salt stress intensifies protein mobilization and/or may impair protein synthesis efficiency due to osmotic and ionic constraints. The markedly higher sensitivity observed in Q2 and Q3 compared with Q1 indicates genotypic differences in nitrogen metabolism and stress adaptation capacity, with Q1 likely maintaining a more stable protein turnover under adverse conditions.

In contrast, vitamin E (α-tocopherol) content exhibited a clear stress-responsive accumulation pattern, especially during germination under control and moderate salinity conditions. The significant increase in all genotypes under non-saline conditions reflects the activation of antioxidant metabolism during seedling establishment, where α-tocopherol plays a key role in protecting cellular membranes from lipid peroxidation associated with increased respiration and metabolic activity [43]. The further stimulation observed in Q1 and Q2 under 300 mM NaCl suggests that moderate salinity acts as an elicitor of antioxidant defense systems, enhancing the synthesis of non-enzymatic antioxidants as part of an adaptive response to oxidative stress. However, the decline in α-tocopherol content under severe salinity (500 mM NaCl) across all genotypes, as also reported by Balakrishnan and Schneider [44], indicates that excessive salt stress overwhelms the antioxidant defense capacity, likely due to excessive reactive oxygen species (ROS) production and disruption of biosynthetic pathways.

In addition to primary metabolism, germination itself induced important modifications in secondary metabolite accumulation. The results revealed strong genotype-dependent and stress-dependent variations in total phenolic content (TPC) and total flavonoid content (TFC), as well as in individual phenolic profiles. Overall, raw seeds exhibited higher baseline levels of phenolic and flavonoid compounds, particularly in Q2, suggesting inherent genetic differences in the capacity for secondary metabolite accumulation among quinoa genotypes. The higher initial TPC and TFC in Q2 may also reflect a greater constitutive antioxidant potential, which could contribute to its previously observed physiological performance under stress conditions.

Germination under non-saline conditions (0 mM NaCl) generally resulted in a reduction in both TPC and TFC across all genotypes compared with raw seeds. This decline may be attributed to the utilization and transformation of phenolic compounds during early metabolic activation, as well as the binding or incorporation of phenolics into structural components of developing tissues. Similar declines during the early stages of germination have been previously reported in quinoa [45] and are commonly linked to the rapid metabolic transition from seed storage reserves toward growth-oriented physiological processes. In contrast, salinity strongly influenced the accumulation of secondary metabolites, including phenolic compounds and flavonoids. These compounds have been shown to play essential roles in quinoa salt tolerance mechanisms due to their antioxidant properties and their capacity to scavenge reactive oxygen species (ROS) generated under stress conditions [46,47]. At moderate salinity, the increase in TFC observed in all seed lots, indicating a stress-induced activation of secondary metabolism and could be interpreted as an adaptive response to salinity-induced oxidative stress, whereby phenolic compounds and flavonoids act as key non-enzymatic antioxidants that protect developing tissues from oxidative damage associated with rapid metabolic activation.

The HPLC-DAD analysis further confirmed these trends at the metabolite level, revealing substantial remodeling of phenolic profiles depending on genotype and salinity level. Under control conditions, germination enhanced the accumulation of several key phenolic and flavonoid compounds, particularly quercetin, rutin, and kaempferol, indicating the activation of the phenylpropanoid pathway during seedling development. This enhancement is associated with the upregulation of phenylpropanoid metabolism and increased activity of phenylalanine ammonia-lyase (PAL), a key enzyme involved in phenolic biosynthesis [48]. Previous studies [49,50] reported that both geographical origin and genotype strongly influence the biosynthesis and accumulation of individual phenolic and flavonoid compounds in quinoa. Similarly, in our case, the observed variability in individual phenolic and flavonoid compounds among quinoa genotypes may be attributed to differences in cultivar origin and environmental adaptation as described in Section 4.

Comparable patterns in TPC and TFC under moderate salinity have been reported among quinoa genotypes from diverse geographical origins, including Andean Peruvian and Chilean accessions, salares landraces, and coastal lowland ecotypes [51,52]. These findings highlight the importance of genotype × environment interactions in shaping secondary metabolite profiles and regulating phenolic metabolism. In the present study, the two-way ANOVA (Table 1) demonstrated that salinity significantly influenced most of the evaluated traits, while genotype effects and, notably, genotype × salinity interactions accounted for a substantial proportion of the observed variability. The particularly strong genotype × salinity effects observed for TFC and TPC suggest that phenolic and flavonoid metabolism was highly responsive to the interaction between genetic background and salt intensity. This supports the strong metabolic plasticity observed among the studied seed lots and suggests that genotype selection is an important determinant of the nutritional and bioactive quality of quinoa seedlings produced under saline conditions.

The coordinated relationships observed among germination, radicle growth, primary metabolites, and antioxidant compounds further suggest that successful seedling establishment under salinity depends on the balance between reserve mobilization, growth, and protective metabolism. In particular, the negative association between growth-related traits and phenolic accumulation may reflect a shift in metabolic allocation from growth toward stress protection under saline conditions. The positive relationships among phenolic compounds, flavonoids, tannins, saponins, and vitamin E also suggest coordinated regulation of antioxidant and secondary metabolic pathways. Beyond these metabolic changes, previous research has highlighted the potential nutritional and metabolic benefits of quinoa germination and fermentation. Evidence from a previous study by Lopes et al. [53] reported that sprouting and fermentation of quinoa contributed to a lower glycemic index of high-carbohydrate diets and were associated with improvements in blood glucose and lipid parameters in Wistar rats. These findings, together with the changes in antioxidant and nutritional compounds observed in the present study, support the potential of quinoa germination as a processing strategy for enhancing its nutritional and functional properties, although further studies are needed to establish the relevance of these effects in humans.

Overall, these findings emphasize the importance of genetic background in determining the nutritional and bioactive quality of quinoa seedlings under salinity and support the potential use of genotype selection to improve the value of quinoa microgreens produced under saline conditions.

Regarding the antinutritional properties of quinoa, saponins are among the most important compounds affecting both nutritional quality and plant defense mechanisms. As characteristic bioactive constituents of quinoa seeds, they contribute to seed bitterness and play a crucial role in protecting plants against biotic and abiotic stresses [54]. Their accumulation is strongly influenced by genotype, environmental conditions, and abiotic factors such as drought and salinity [47]. In the present study, germination resulted in a substantial reduction in saponin levels across all varieties, confirming that soaking and sprouting are effective approaches for decreasing saponin concentration and improving quinoa palatability. This reduction may be attributed to the leaching of water-soluble saponins during soaking, as well as their metabolic degradation and utilization during seedling development, as previously reported by Rani et al. (2026) [55]. Furthermore, the decline may be associated with the hydrolysis of triterpenoid glycosides and the subsequent use of their degradation products in cellular metabolism during seedling establishment [56]. The particularly pronounced reduction observed in genotype Q3 suggests a greater capacity for saponin mobilization or degradation during germination. Salinity also influenced saponin accumulation, and the increased saponin content observed under moderate salinity in genotype Q2 may reflect an adaptive metabolic response to salt stress. Given the reported roles of saponins in membrane stabilization and stress-related signaling pathways, their accumulation could contribute to enhanced stress tolerance and indicate a greater physiological responsiveness of this genotype to saline conditions.

The correlation analysis provides evidence of coordinated relationships between saponin accumulation and several primary and secondary metabolites (Figure 4). The positive associations of saponin content with starch, total phenolic content, protein content, tannins, and total flavonoid content suggest that saponin accumulation may be linked to the synthesis and storage of both reserve compounds and secondary metabolites. In contrast, the negative correlations observed between saponin content, germination percentage and root length may indicate a potential trade-off between the accumulation of defense-related metabolites and early seed establishment. This relationship could be associated with the biological activity of saponins, which may influence membrane properties and cellular processes during germination. Tannins, which are polyphenolic compounds with recognized roles in ROS scavenging and cellular protection, were also positively correlated with saponins and vitamin E, suggesting a coordinated accumulation of antioxidant-related metabolites that may contribute to protection against oxidative stress. Moreover, the increased tannin content observed during germination, particularly in genotype Q1 under control and moderate salinity conditions, indicates enhanced activation of the phenylpropanoid pathway during early seedling development and increased synthesis of phenolic polymers involved in antioxidant defense. However, the decreased tannin content recorded in Q1 and Q2 under severe salinity suggests a possible inhibition of phenolic biosynthesis or a reallocation of metabolic resources toward essential survival and stress-adaptation processes under elevated salinity stress.

Based on the observations and analyses presented above, the present study indicates that environmental conditions experienced during seed development can substantially influence seed quality, germination behavior, and subsequent seedling responses [57]. As discussed above, an important consideration when interpreting the present results is the origin and propagation history of the seed material. Although Q2 and Q3 belong to the same *Amarilla sacaca* variety, their contrasting responses, particularly for some germination, growth, and metabolic parameters, may reflect not only their common genetic background but also maternal and environmental effects associated with seed production. However, the available information on their propagation environments does not allow the relative contributions of genetic, maternal, and environmental factors to be quantitatively separated. This represents an important limitation of the present study and should therefore be considered when interpreting the differential responses of seed lots to salinity. Future studies using plant material with a common genetic background propagated under standardized environmental conditions, or replicated multi-environment trials, will be necessary to disentangle genetic effects from maternal and environmental influences and to confirm the stability of the observed responses to salinity. Such approaches would provide a stronger basis for determining the extent to which seed-production conditions contribute to variation in salinity tolerance during quinoa germination and early seedling development.

## 4. Materials and Methods

### 4.1. Seeds Collection, Germination and Salt Treatments

Three quinoa (*Chenopodium quinoa* Willd.) seed lots representing two varieties were used in this study: Regalona (Q1) and *Amarilla sacaca* (Q2 and Q3). Regalona, originally from southern Chile, is characterized by high yield, a high harvest index, large 1000-kernel weight, and good product homogeneity and quality [58]. *Amarilla sacaca*, originally from Peru, is characterized by its orange-colored seeds and relatively long growth cycle (170–180 days). Regalona (Q1) and *Amarilla sacaca* (Q2) were cultivated and multiplied in southern Tunisia under Saharan conditions and saline soil, whereas *Amarilla sacaca* (Q3) was cultivated and multiplied in southern Algeria under high-temperature conditions. Thus, Q2 and Q3 represent the same quinoa variety, *Amarilla sacaca*, but were propagated under different geographical and environmental conditions. Regalona seeds were originally provided by the USDA International Seed Bank, whereas the original *Amarilla sacaca* seeds were supplied by the Technical Institute for the Development of Desert Agriculture (ITDAS), Algeria. The three seed lots were subsequently used to evaluate germination and seedling responses to NaCl-induced salinity.

Prior to the germination assay, seeds were surface-sterilized by soaking in a 20% (*v*/*v*) sodium hypochlorite solution for 5 min, followed by thorough rinsing with distilled water (five times). Germination tests were then conducted under three treatments: control (distilled water) and NaCl solutions at 300 and 500 mM. For each treatment, 300 seeds were used and distributed into six Petri dishes (50 seeds per dish) lined with a double layer of filter paper moistened with the respective NaCl solutions (0, 300, or 500 mM). The Petri dishes were incubated in a germination chamber maintained at 22 ± 1 °C, 70 ± 5% relative humidity, and a 16 h photoperiod with a light irradiance of 150 μmol m^−2^ s^−1^. Germination was monitored every 2 h throughout the experimental period, and the number of germinated seeds was recorded. Once the final germination percentage was reached, with no additional germinated seeds observed, the seedlings were allowed to develop for an additional four days. After this period, the seedlings were harvested separately and analyzed for their nutritional and antinutritional properties.

### 4.2. Germination Analysis

The final germination percentage (%) represents the proportion of germinated seeds relative to the total number of seeds sown in each Petri dish at the end of the experimental period and was calculated using the following formula:

$$\mathrm{Germination}\;\mathrm{Percentage}\;(\%)\;=\;\frac{\mathrm{Final}\;\mathrm{number}\;\mathrm{of}\;\mathrm{germinated}\;\mathrm{seeds}}{\mathrm{Total}\;\mathrm{number}\;\mathrm{of}\;\mathrm{seeds}}\times 100$$

Time to 50% germination (T_50_) was also evaluated to determine the time required for 50% of the final germination to be achieved and to assess the speed of germination within the seed population. T_50_ was calculated using the mathematical formula proposed by Hao et al. (2017) [59]:

$$\mathrm{Time}\;\mathrm{to}\;50\%\;\mathrm{Germination}\;(T_{50})\;=\;\frac{\mathrm{ti}+(\frac{N}{2}-n_{i})(t_{j}-t_{i})}{\left(n_{i}-n_{j}\right)}$$

where *N* is the final number of germinated seeds, (*n_i_*) and (*n_j_*) are the cumulative numbers of seeds germinated at two consecutive observation times (*t_i_*) and (*t_j_*), respectively, such that *n_i_* < *N/2* < *n_j_*. Thus, *t_i_* and *t_j_* represent the two consecutive observation times between which 50% of the final germination is reached, rather than the initial observation time and the exact time at which 50% germination occurs.

The radicle and shoot lengths and fresh and dry weight of the seedlings were also measured at the end of the experimental period.

### 4.3. Nutritional Properties

#### 4.3.1. Protein Extraction and Quantification

Total soluble proteins concentration was assessed following the method described by Bradford (1976) [60]. An aliquot of 0.5 g of finely ground, lyophilized raw seeds or seedlings was homogenized in cold Tris–HCl extraction buffer (pH 8.0) containing 10 mM EDTA, 50 mM KCl, 20 mM MgCl2, 0.5 mM phenylmethylsulfonyl fluoride (PMSF), 1 mM dithiothreitol (DTT), 0.1% (*v*/*v*) Triton X-100, and 10% (*w*/*w*) polyvinylpyrrolidone (PVP). The homogenates were centrifuged at 14,000× *g* for 15 min at 4 °C, and the resulting supernatants were collected, mixed with Bradford reagent and then incubated for 5 min. Absorbance was measured at 595 nm using a spectrophotometer, and protein concentration was determined by interpolation from a calibration curve prepared using bovine serum albumin (BSA) as the standard. The results were expressed as mg/g dry weight (DW).

#### 4.3.2. Soluble Sugars Content

Total soluble sugars were determined according to the method described by Yemm and Willis (1954) [61]. A 0.25 g of portion of finely ground, lyophilized raw seeds or seedlings was homogenized in 5 mL of 70% ethanol and centrifuged at 8000 rpm for 10 min at 15 °C. An aliquot of 200 µL of the resulting supernatant was transferred into glass test tubes and mixed with 1 mL of anthrone reagent. The mixture was vortexed and incubated in a boiling water bath at 100 °C for 10 min. The reaction was stopped by immediately cooling the tubes on ice. Absorbance was measured at 625 nm using a spectrophotometer. Total soluble sugar concentration was determined by interpolation from a calibration curve prepared using D-glucose as the standard and expressed as mg/g DW.

#### 4.3.3. Starch Content

Starch content was determined using the iodine colorimetric method previously described by Vainio (1968) [62] and subsequently modified by Bahdanovich et al. (2022) [63]. Briefly, 0.1 g of finely ground, lyophilized raw seeds or seedlings was extracted with 10 mL of a mixture of 1 N HCl and 1 N KOH and heated at 100 °C for 15 min. The mixture was then sonicated for 10 min to facilitate starch extraction, after which it was centrifuged and the supernatant was collected. An aliquot of 50 µL of the resulting extract was mixed with 0.1 mL of KI/I_2_ solution, and the volume was adjusted to 5 mL with distilled water. Absorbance was measured at 720 nm using a spectrophotometer. Starch concentration was determined by interpolation from a calibration curve prepared using known concentrations of soluble starch under the same reaction conditions and expressed as mg/g DW.

#### 4.3.4. Vitamin E Content Determination

Vitamin E (α-tocopherol) content was estimated according to the colorimetric method described by Backer and Davis (1996) [64], based on the Emmerie–Engel reaction. Briefly, 0.25 g of finely ground, lyophilized raw seeds or seedlings was homogenized in a mixture of petroleum ether and ethanol (2:1.6, *v*/*v*). The homogenate was centrifuged at 10,000 rpm for 20 min, and an aliquot of 1 mL of the resulting supernatant was mixed with 0.2 mL of 2,2-dipyridyl solution (2%) and 0.5 mL of FeCl_3_ solution (0.2%). The mixture was thoroughly vortexed and incubated in the dark for 5 min to allow the development of the red-colored complex. Absorbance was measured at 520 nm using a spectrophotometer. Vitamin E content was determined by interpolation from a calibration curve prepared using known concentrations of α-tocopherol and expressed as mg/g DW.

### 4.4. Bioactive Compound and Antinutritional Factors Analysis

#### 4.4.1. Preparation of the Hydroalcoholic Extract

The hydroalcoholic extract was prepared according to the method described by Miranda et al. (2023) [65] with slight modifications. Briefly, 0.5 g of raw seeds or seedlings was crushed and macerated in 70% ethanol. The mixture was then subjected to sonication using an ultrasonic cleaner (sweep technology BPAC) for 30 min at 40 °C to enhance the extraction of bioactive compounds. The resulting extract was filtered and concentrated using rotary evaporator (Z603716 Büchi^®^ rotary evaporator Model R-205). The concentrated extract was subsequently lyophilized using a Christ Beta 2–8 LD plus Lyophilizer to obtain the crude extract. The dried extract was weighed, and distilled water was added to prepare a final concentration of 10 mg/mL. The obtained extract was later used for the determination of secondary metabolites, including flavonoids, polyphenols, tannins, and saponins.

#### 4.4.2. Total Phenolic Content (TPC)

Total phenolic content was quantified using the Folin–Ciocalteu method according to Singleton and Rossi (1965) [66]. Briefly, 125 µL of sample was mixed with 500 µL of distilled water and 125 µL of Folin–Ciocalteu reagent (1:9, *v*/*v*). Then, 1.25 mL of sodium carbonate solution was added, and the mixture was incubated in the dark at room temperature for 90 min. Absorbance was measured at 760 nm using a spectrophotometer. TPC was determined by interpolation from a calibration curve prepared using gallic acid as the standard and expressed as mg GAE/g DW.

#### 4.4.3. Total Flavonoids Content (TFC)

Total flavonoid content (TFC) was determined using the aluminum chloride colorimetric method previously described by Chang et al. (2002) [67]. Briefly, 400 µL of the sample was mixed with 120 µL of sodium nitrite solution (5%), followed by the addition of 120 µL of aluminum chloride solution (10%) and 800 µL of sodium hydroxide (1 M). The mixture was thoroughly mixed, and absorbance was measured at 510 nm using a spectrophotometer. TFC was determined by interpolation from a calibration curve prepared using quercetin as the standard and expressed as mg QE/g DW.

#### 4.4.4. Tannins Content Estimation

Total tannins content was measured using the vanillin-HCl method according to Broadhurst and Jones (1978) [68]. Briefly, 0.2 mL of sample was mixed with 1 mL of vanillin reagent prepared in a mixture of HCl and Acetic acid (*v*/*v*). The mixture was incubated in a water bath at 30 °C for 20 min, and absorbance was recorded at 410. Tannin content was determined from a catechin calibration curve and expressed as mg CE/g DW.

#### 4.4.5. Saponin Quantification

Saponin content was quantified using a colorimetric assay based on the protocols of Patel et al. (2012) [69] and Ncube et al. (2011) [70]. The samples (raw seeds or seedlings) were first mixed with ethanol 95%, followed by the addition of half the sample volume of water, to achieve a final solution concentration of 10 mg/mL. To the obtained extract (125 µL) was added 125 μL of vanillin 0.8% (*w*/*v*) in ethanol and 1.25 mL of 72% (*v*/*v*) sulfuric acid. The mixture was incubated at 60 °C for 10 min, and then cooled on ice bath. Total saponin content was determined by interpolation from a calibration curve prepared using 100% pure saponin as the standard and expressed as mg/g DW.

### 4.5. HPLC- DAD Analysis of Phenolic Acids and Flavonoids

The identification of phenolic compounds in quinoa seeds was evaluated using HPLC- DAD method as previously described by Paśko et al. (2008) [71]. The extracts were analyzed using a high-performance liquid chromatography system equipped with a diode array detector (HPLC–DAD) (Agilent 1260, Agilent Technologies, Santa Clara, CA, USA), consisting of a vacuum degasser, an autosampler, and a binary pump capable of 400 bar maximum pressure. Separation was performed on a reversed-phase C18 analytical column Zorbax Eclipse XDB C18, 4.6 × 100 mm, 3.5 μm particle size (Agilent Technologies, Santa Clara, CA, USA). The column temperature was maintained at 23 °C, and the injected sample volume was 2 μL for standard solutions and 50 μL for extract samples. The mobile phase consisted of phase A: acetonitrile and phase B: Milli-Q water with 0.1% formic acid, with a flow rate of 0.4 mL/min. The gradient elution program was optimized as follows: 0–5 min, 10–20% A; 5–10 min, 20–30% A; 10–15 min, 30–50% A; 15–20 min, 50–70% A; 20–25 min, 70–90% A; 25–30 min, 90–50% A; 30–45 min, return to initial conditions. The DAD detector was set to scan 200–400 nm. Extracts were injected using the autosampler at 50 min intervals, with each analysis lasting 50 min. Under these conditions, apigenin, vitexin, isovitexin, orientin, isoorientin, kaempferol, quercetin, rutin, myricetin, morin, naringin, hesperidin, neohesperidin, gallic acid, cinnamic acid, vanillic acid, caffeic acid, ferulic acid, p-coumaric acid, p-hydroxybenzoic acid, and syringic acid were analyzed as standard solutions in ethanol (0.1 mg/mL). Each standard solution was injected twice, and the average retention times (tR) were recorded. Compounds in the seed and sprout extracts were identified by comparison of tR and UV spectra with those of standards. Quantification was achieved using calibration plots constructed from injections of individual standard solutions at volumes ranging from 5 to 30 μL in 5 μL increments, which showed linear responses over the investigated range. All extract analyses were performed in three independent replicates, and the concentrations of phenolic acids and flavonoid compounds are presented as mean ± SD.

### 4.6. Statistical Analyses

All obtained data were analyzed using OriginLab software (OriginPro 2020, SR1). Mean values and standard error (SE) were calculated from six independent replicates for physiological and biochemical parameters and from three independent replicates for the phenolic compounds identified by HPLC-DAD analysis. Data were subjected to a two-way factorial ANOVA to evaluate the effects of seed lot (Q1, Q2, and Q3), NaCl concentration, and their interaction. Significant differences among means were assessed using Duncan’s multiple range test at *p* ≤ 0.05. Pearson’s correlation analysis was conducted to assess the strength and direction of linear relationships among the investigated parameters. Correlation coefficients (r) ranged from −1 (perfect negative correlation) to 1 (perfect positive correlation), with 0 indicating no linear correlation.

## 5. Conclusions

This study provides an integrated assessment of the effects of NaCl-induced salinity on germination, early seedling development, and biochemical composition in three quinoa seed lots representing two varieties: Regalona (Q1) and *Amarilla sacaca* (Q2 and Q3), highlighting strong genotype-dependent variability in both germination performance and metabolic regulation. Salinity, particularly at high levels, obviously inhibited germination capacity and early seedling growth, likely due to the combined effects of osmotic stress and ion toxicity, which can impair reserve mobilization and embryo development. Among the tested seed lots, Q2 (*Amarilla Sacaca*) generally maintained a higher germination rate under saline conditions and showed comparatively smaller changes in several biochemical parameters, including those related to primary metabolism and antioxidant responses. This suggests that Q2 was better able to maintain metabolic homeostasis under salinity during early development. However, because Q2 and Q3 belong to the same genotype *Amarilla sacaca* but originated from different propagation environments, these differences should not be interpreted solely as evidence of genetic variation and may also reflect differences in seed physiological quality and maternal or environmental effects.

Beyond germination traits, salinity and germination significantly reshaped the nutritional and phytochemical composition of quinoa seeds. Germination generally promoted reserve mobilization, as evidenced by starch degradation and protein turnover, while simultaneously inducing dynamic changes in soluble sugars and antioxidant compounds. Moderate salinity acted as an elicitor of secondary metabolism, enhancing the accumulation of phenolic compounds, flavonoids, tannins, and vitamin E, thereby strengthening antioxidant defenses. However, severe salinity disrupted these metabolic adjustments, indicating that excessive stress overwhelms biosynthetic pathways and limits metabolic plasticity. In contrast, the maintenance of higher antioxidant and phenolic levels in genotype *Amarilla sacaca* variety under stress conditions reflects genotype-specific strategies for oxidative stress mitigation and metabolic resilience.

From a broader perspective, these results suggest promising ways to use quinoa as a functional microgreen crop, where controlled abiotic stress can enhance nutritional and antioxidant profiles. Future research should validate these findings in controlled systems, investigate molecular mechanisms behind genotype-specific stress responses, and determine optimal stress levels that improve yield and bioactive compounds without reducing germination. This could support quinoa-based functional foods and more sustainable production under increasing soil salinity.

## Figures and Tables

**Figure 1:**
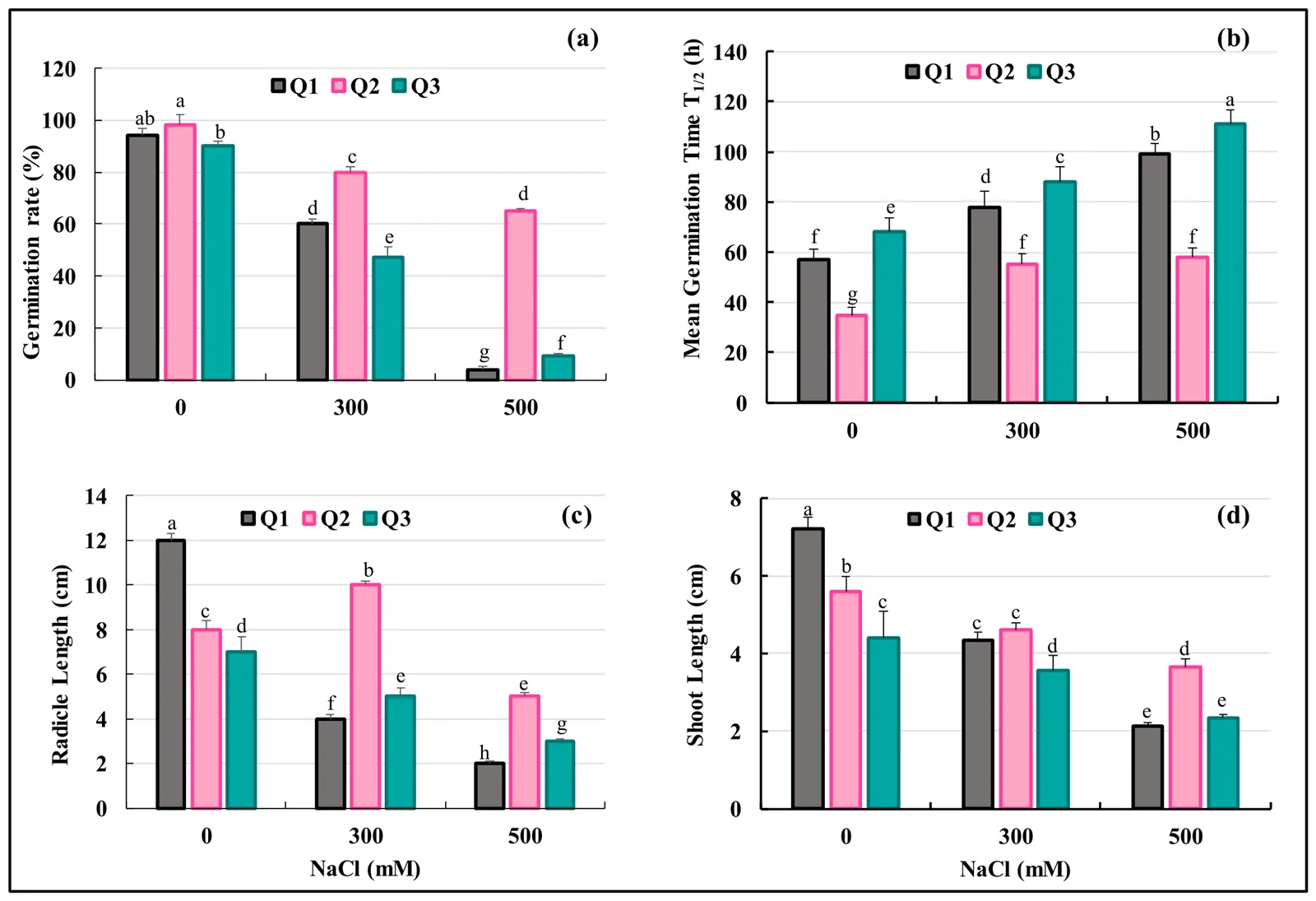
Effect of NaCl-induced salinity on seed germination and early seedling growth. (**a**) Germination rate (%), (**b**) mean germination time (T_1/2_, h), (**c**) radicle length (cm) and (**d**) shoot length (cm) of three quinoa seed lots (Q1, Q2, Q3) cultivated under different NaCl concentrations (0, 300, and 500 mM). Data are presented as means ± SD (*n* = 6). Bars sharing the same letter are not significantly different at *p* < 0.05, according to Duncan’s multiple range test.

**Figure 2:**
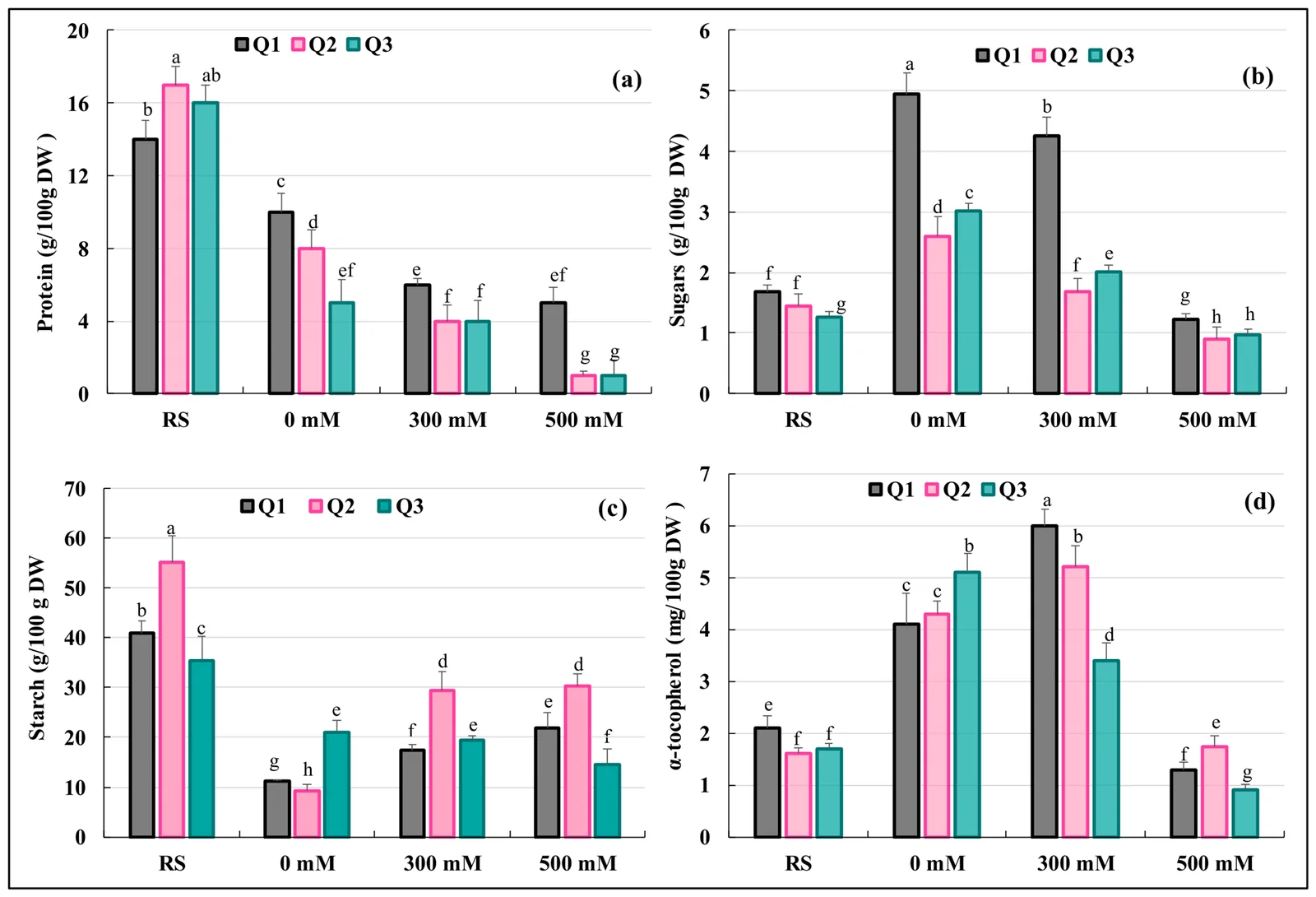
Protein content (**a**), soluble sugar (**b**), starch content (**c**) and α-tocopherol (**d**), in raw seeds (RS) and seedlings cultivated under different NaCl concentrations (0, 300, and 500 mM) of three quinoa seed lots (Q1, Q2, Q3). Data are presented as means ± SD (n = 6). Bars sharing the same letter are not significantly different at *p* < 0.05, according to Duncan’s multiple range test.

**Figure 3:**
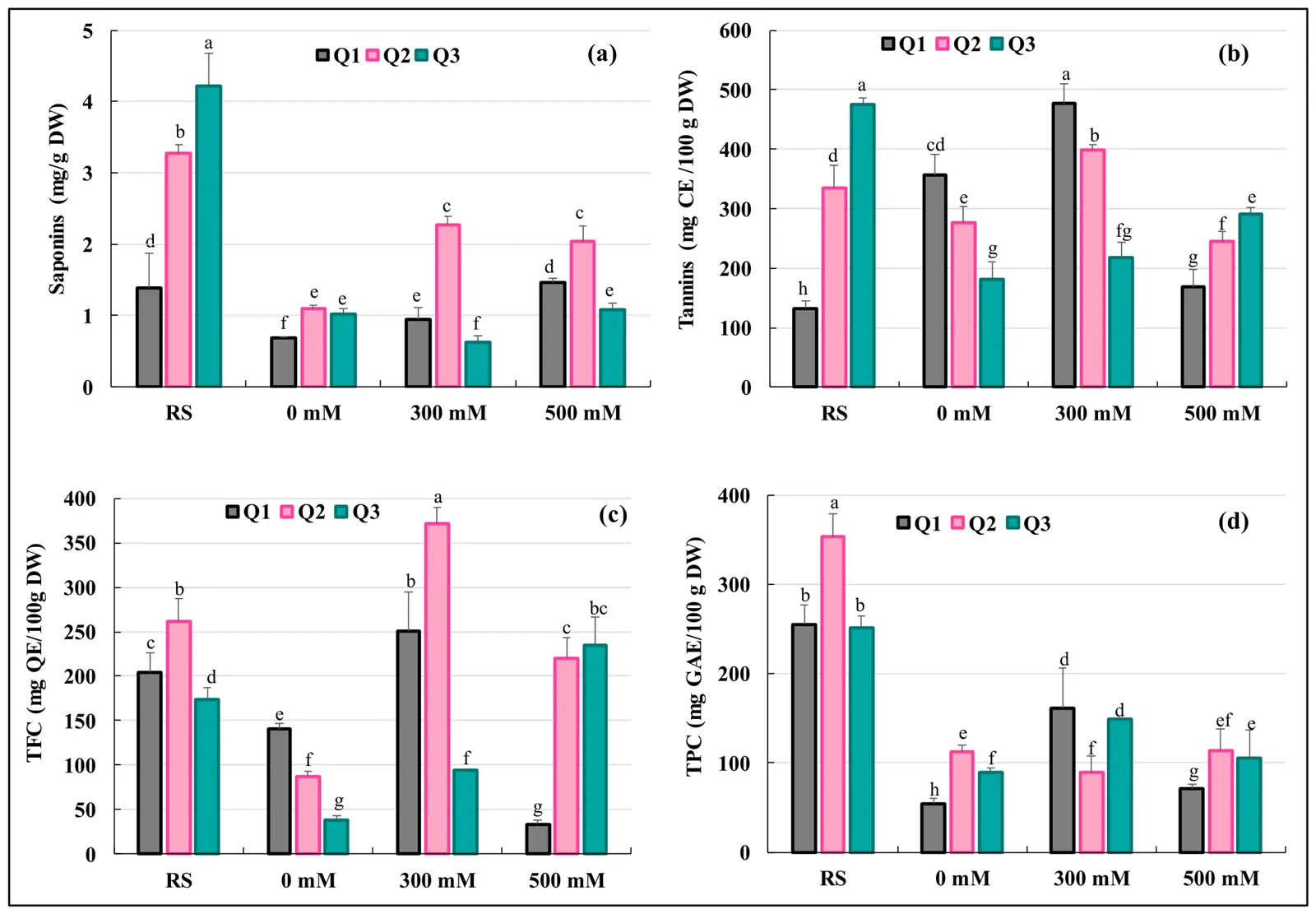
Saponin content (**a**), tannins (**b**), total flavonoid content (TFC) (**c**), and total phenolic content (TPC) (**d**) in raw seeds (RS) and seedlings cultivated under different NaCl concentrations (0, 300, and 500 mM) of three quinoa seed lots (Q1, Q2, Q3). Data are presented as means ± SD (n = 6). Different letters indicate statistically significant differences among treatments (*p* < 0.05).

**Figure 4:**
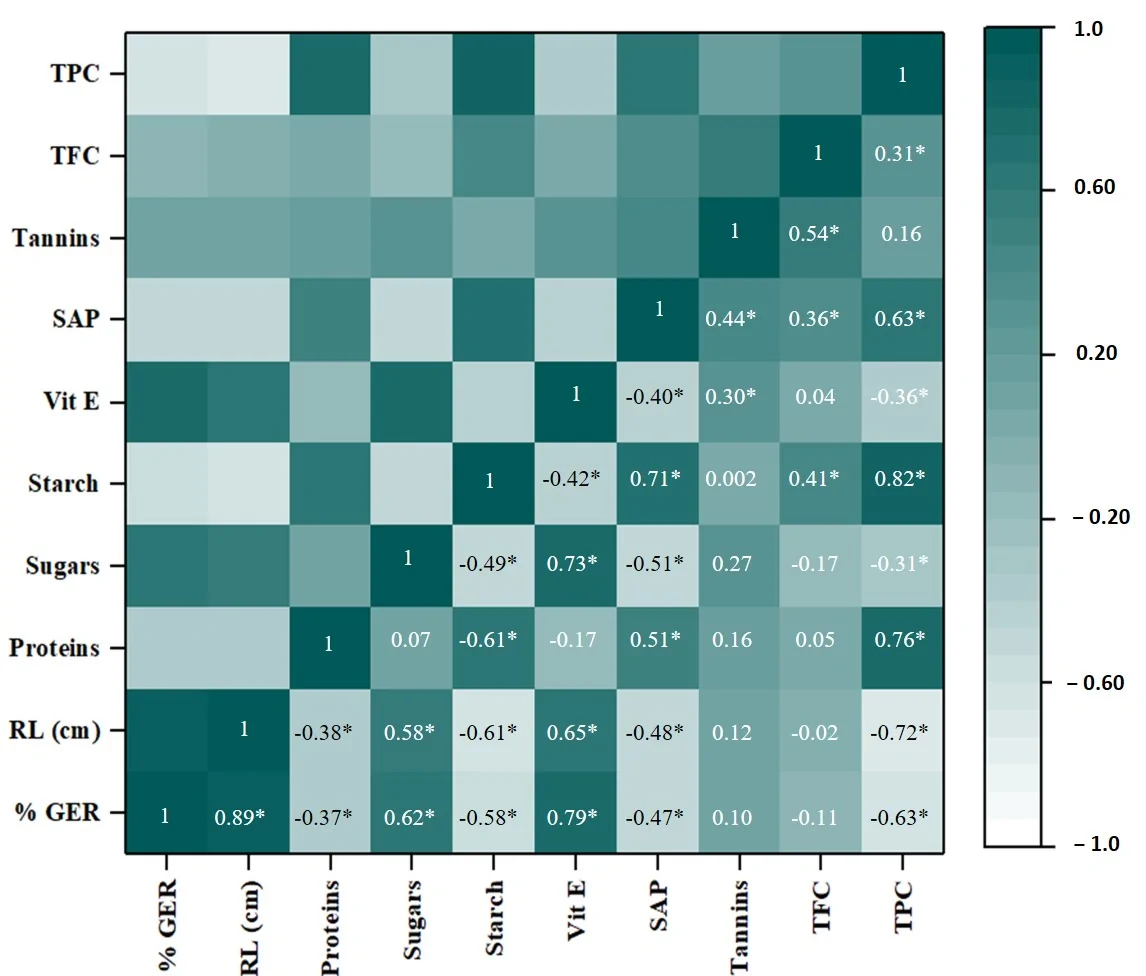
Correlation matrix based on Pearson correlation coefficient showing relationships among variables measured in seedlings of quinoa genotypes cultivated under different NaCl concentrations. The colors represent the correlation, with green being more positive, and white more negative. Significance is given as * (*p* < 0.05).

**Table 1:** Summary of two-way ANOVA for different parameters of germination, metabolic and bioactives compounds in RS and seedling of quinoa genotypes cultivated under different salinity conditions.

Trait	Source of Variation	*F* Value	*p* Value	Sig.
Germination rate	Conditions (E)	78.39	0.00045	***
	Genotype (G)	19.99	7.93 × 10^−7^	***
	G × E	615.0	0.0012	**
Radicle length	Conditions (E)	51.89	3.50 × 10^−14^	***
	Genotype (G)	5.04	0.01085	***
	G × E	45.47	2.33 × 10^−15^	***
Starch	Conditions (E)	389.46	0.025	*
	Genotype (G)	72.38	2.43 × 10^−13^	***
	G × E	40.81	1.28 × 10^−14^	***
Sugars	Conditions (E)	23.16	3.77 × 10^−9^	***
	Genotype (G)	6.56	0.00315	**
	G × E	34.35	1.77 × 10^−13^	***
Proteins	Conditions (E)	1132.9	0.033	*
	Genotype (G)	107.16	6.66 × 10^−16^	***
	G × E	35.29	1.18 × 10^−13^	***
Vit. E	Conditions (E)	93.67	0.045	*
	Genotype (G)	0.48	0.019	*
	G × E	141.48	0.026	*
Tannins	Conditions (E)	3.48	0.0236	*
	Genotype (G)	31.78	1.12 × 10^−8^	***
	G × E	615.03	0.012	*
Saponin	Conditions (E)	16.82	2.00 × 10^−7^	***
	Genotype (G)	4.30	0.01954	*
	G × E	215.55	0.0112	*
TFC	Conditions (E)	7.91	2.48 × 10^−4^	***
	Genotype (G)	5.44	0.00762	**
	G × E	4719.5	0.0048	**
TPC	Conditions (E)	91.27	01.56 × 10^−5^	***
	Genotype (G)	0.515	0.089	*
	G × E	2312.1	0.0035	**

Sig. = significance. *, *p* < 0.05; **, *p* < 0.01; ***, *p* < 0.001.

**Table 2:** Content of individual phenolic and flavonoid compounds under different conditions. Data (mg/100 g DW) are presented as mean ± SD and represent means of three independent measurements (n = 3). Different lowercase letters indicate significant differences among seed lots (Q1, Q2, and Q3) under RS, 0, 300, and 500 mM NaCl conditions for each compound (one-way ANOVA followed by Duncan’s test, *p* < 0.05). n.d. = not detected; RS = raw seeds.

Conditions	Genotypes	Gallic Acid (Peak 1)	Syringic Acid (Peak 2)	Rutin (Peak 3)	Quercetin (Peak 4)	Vanillic Acid (Peak 5)	Protocatechuic Acid (Peak 6)	Kaempferol (Peak 7)	Caffeic Acid (Peak 8)
RS	Q1	0.84 ± 0.11 c	2.70 ± 0.22 c	0.51 ± 0.06 d	1.30 ± 0.15 f	22.2 ± 2.60 a	n.d.	n.d.	n.d.
RS	Q2	0.77 ± 0.09 cd	2.69 ± 0.21 c	n.d.	4.10 ± 0.48 e	n.d.	53.00 ± 6.22 a	n.d.	10.1 ± 1.18 b
RS	Q3	0.88 ± 0.12 c	3.10 ± 0.34 b	0.23 ± 0.03 e	6.60 ± 0.77 cd	13.2 ± 1.55 c	65.00 ± 6.33 a	0.14 ± 0.02 c	13.0 ± 1.52 a
0 mM	Q1	0.99 ± 0.12 b	3.60 ± 0.41 b	0.81 ± 0.09 b	6.30 ± 0.74 cd	20.1 ± 2.35 a	n.d.	n.d.	5.7 ± 0.67 c
0 mM	Q2	0.74 ± 0.09 d	3.50 ± 0.32 b	n.d.	10.10 ± 1.18 b	n.d.	54.5 ± 6.38 a	n.d.	12.1 ± 1.42 a
0 mM	Q3	0.93 ± 0.11 b	4.21 ± 0.32 a	0.55 ± 0.06 d	14.60 ± 1.71 a	12.2 ± 1.43 c	63.5 ± 7.44 a	0.72 ± 0.08 a	14.4 ± 1.69 a
300 mM	Q1	1.51 ± 0.18 a	3.95 ± 0.35 ab	2.22 ± 0.18 a	7.89 ± 0.92 c	26.8 ± 4.14 a	n.d.	n.d.	11.4 ± 1.33 b
300 mM	Q2	0.73 ± 0.09 d	3.40 ± 0.22 b	n.d.	11.2 ± 1.31 b	n.d.	26.3 ± 3.08 b	n.d.	11.1 ± 1.30 b
300 mM	Q3	0.92 ± 0.11 b	4.10 ± 0.41 a	0.87 ± 0.08 b	13.3 ± 1.56 a	17.7 ± 2.07 b	32.2 ± 3.71 b	0.71 ± 0.08 a	12.2 ± 1.43 ab
500 mM	Q1	0.42 ± 0.05 e	0.90 ± 0.11 e	0.72 ± 0.05 c	3.15 ± 0.37 e	14.5 ± 1.70 c	n.d.	n.d.	4.2 ± 0.49 d
500 mM	Q2	0.24 ± 0.03 f	0.88 ± 0.10 e	n.d.	5.4 ± 0.63 d	n.d.	11.1 ± 1.30 d	n.d.	3.2 ± 0.37 d
500 mM	Q3	0.44 ± 0.05 e	1.21 ± 0.14 d	0.52 ± 0.07 d	5.7 ± 0.44 d	6.6 ± 0.77 d	14.5 ± 1.70 c	0.21 ± 0.02 b	4.5 ± 0.53 c

## Data Availability

The raw data supporting the conclusions of this article will be made available by the authors on request.

## Abbreviations

The following abbreviations are used in this manuscript:

RS	Raw seeds
SAP	Saponins
CE	Catechin
GAE	Gallic Acid
ROS	Reactive Oxygen Species
TPC	Total Phenolic Content
TFC	Total Flavonoid Content
QE	Quercetin
DW	Dry weight
FW	Fresh weight
T_1/2_: T50	Mean germination time
RL	Root length
Vit. E	Vitamin E
BSA	Bovine Serum Albumin
